# Stochastically Timed Competition Between Division and Differentiation Fates Regulates the Transition From B Lymphoblast to Plasma Cell

**DOI:** 10.3389/fimmu.2018.02053

**Published:** 2018-09-10

**Authors:** Jie H. S. Zhou, John F. Markham, Ken R. Duffy, Philip D. Hodgkin

**Affiliations:** ^1^Immunology Division, The Walter and Eliza Hall Institute of Medical Research, Parkville, VIC, Australia; ^2^Department of Medical Biology, The University of Melbourne, Parkville, VIC, Australia; ^3^Victoria Research Laboratory, National ICT Australia, The University of Melbourne, Parkville, VIC, Australia; ^4^Hamilton Institute, Maynooth University, Maynooth, Ireland

**Keywords:** B cells, anti-CD40 stimulation titration, fate regulation, lineage priming, competing stochastic timers

## Abstract

In response to external stimuli, naïve B cells proliferate and take on a range of fates important for immunity. How their fate is determined is a topic of much recent research, with candidates including asymmetric cell division, lineage priming, stochastic assignment, and microenvironment instruction. Here we manipulate the generation of plasmablasts from B lymphocytes *in vitro* by varying CD40 stimulation strength to determine its influence on potential sources of fate control. Using long-term live cell imaging, we directly measure times to differentiate, divide, and die of hundreds of pairs of sibling cells. These data reveal that while the allocation of fates is significantly altered by signal strength, the proportion of siblings identified with asymmetric fates is unchanged. In contrast, we find that plasmablast generation is enhanced by slowing times to divide, which is consistent with a hypothesis of competing timed stochastic fate outcomes. We conclude that this mechanistically simple source of alternative fate regulation is important, and that useful quantitative models of signal integration can be developed based on its principles.

## Introduction

Increased understanding of the regulation of cell differentiation, division and death is crucial in many fields of biology (1–5). While population-level consistency in the proportion of cells taking on distinct fates has long-since been observed, advancing technologies that enable direct observations of individual cells and their lineages reveal significant heterogeneity (6–14). In order to manipulate population-level fate allocation, determining the primary drivers of this cell-level heterogeneity is an essential precursor to designing interventions, and so the search for the sources of heterogeneity has been a topic of much recent research.

B lymphocytes are an essential component of the immune response and provide a useful model for assessing methods of fate control. During activation, they integrate signals from multiple sources that modify the resulting cell response by altering lifespan, the type of antibody made, the speed of cell proliferation, and the rate of development into antibody secreting plasma cells (15). T cells provide one important source of signals that influence the B cell (16, 17). During an immune response, antigen captured by the B cell is presented to reactive T cells that are, in turn, induced to express CD40L on their surface. This ligand engages the constitutively expressed receptor CD40 found on the B cell surface. CD40 stimulation alone can activate and promote B cell proliferation, but its impact is amplified by T cell derived cytokines, such as IL-4 and IL-5, that further shape fate changes including isotype switching and the rate of development into Antibody Secreting Cells (ASCs) (18). Importantly for quantitative studies, a CD40 agonist and cytokines can replace the T cell, making it an excellent model system for studying the impact of variations in signals on fate outcomes *in vitro*.

Activated lymphocytes vary in the times they take to divide and, in culture, are usually found spread across multiple generations. Notwithstanding that, numerous studies report that the greater the number of divisions cells have experienced, the more likely they are to have undergone a change, regardless of time from stimulation. For example, isotype switching is linked to division, and is influenced by the concentration of switch-inducing cytokines (19–21). Similarly, the development into ASC also has been reported to be promoted by progressive passage through division cycles, and this likelihood, in turn, is modulated by the concentration of cytokine delivered signals (18, 22). These studies proposed that alternative division-linked cell changes, such as switching and development to ASC, could arise as the combination of a series of independent fate decisions underway in each cell (18, 20, 23–25).

This hypothesis of independent fate competition was evaluated and extended upon by Duffy et al. (26), by assessing data taken from experiments where individual B cells that had undergone given numbers of divisions, as determined by CTV staining, were sorted by flow cytometry and subsequently followed with long-term imaging. Once these cells were observed to divide, their sibling offspring were examined with times to divide, to die, to differentiate to ASC, and to antibody isotype-switch from last mitosis recorded. Probabilistic analysis established that the complex array of heterogeneous fate outcomes and times to fates were consistent with a simple hypothesis where, within each single cell, times to each fate (isotype switching, differentiation, death, and division) were selected independently from a probability distribution and behaved in competition with each other, such that short times to fate censor later fate outcomes (26). Sibling fates, however, had significantly greater commonality than unrelated cells of the same generation, indicating a substantial element of familial lineage priming.

As external regulators are known to influence the proportions of cells assuming fates at a population level, we reasoned that investigation of their impact at the single cell level would provide additional discriminating insight. Here we examine the cell-level impact of changing CD40 stimulation strength on ASC development. This analysis suggests that CD40 has no direct impact on differentiation rate or asymmetric fate changes, but exerts its influence by altering the time to divide distribution, thereby regulating the proportion of cells that differentiate as the result of alterations to the inherent cellular competition.

## Materials and methods

### Mice

Blimp-1-GFP reporter mice on a C57BL/6 background (22) were bred and maintained in specific pathogen free conditions at the Walter and Eliza Hall Institute (WEHI) animal facility according to institutional guidelines. All experiments were approved by the WEHI Animal Ethics Committee. Ten-week-old female reporter (Blimp-1^+/GFP^) and wild type (Blimp-1^+/+^) mice were used for the flow cytometry and filming experiments.

### Cell isolation and labeling

Naïve, resting B cells were isolated from murine spleens using a discrete Percoll (GE Healthcare, cat#17089101) density gradient (50/65/80%, cells collected from 65/80% interface), followed by purification using magnetic beads (negative selection, mouse B cell isolation kit, Miltenyi Biotech cat#130-090-862). Enriched cells were verified to be >98% B220^+^ and CD19^+^ by flow cytometry. Cells were labeled with CellTrace Violet (Invitrogen, cat#C34557) at 7.5 μM final concentration, with 10^7^ cells/mL in phosphate buffered saline containing 0.1% bovine serum albumin (PBS/0.1%BSA), and incubated in a 37°C water bath for 20 min. Cells were washed twice with cold culture medium prior to culture.

### Cell culture

For flow cytometry and for bulk cultures, cells were cultured in “B cell medium,” made from Advanced RPMI 1640 (Gibco cat#12633-012) supplemented with 5% fetal calf serum (Gibco, cat#10099-141, Australian origin), 10 mM HEPES (Gibco, cat#15630-130), 2 mM GlutaMAX (Gibco, cat#35050-061), 10 U/mL penicillin, 100 μg/mL streptomycin (Penicillin/Streptomycin, Gibco, cat#15140-148), and 50 μM 2-mercaptoethanol (2-ME, Sigma-Aldrich, cat#M7522). For filming, cells were cultured in phenol red-free Advanced RPMI 1640 (Gibco custom order) with the same supplements. Imaged cells were stimulated with 1,000 U/mL IL-4 (WEHI), and 10, 2.5, or 0.625 μg/mL anti-CD40 antibody (1C10, WEHI Antibody Facility), and incubated at 37°C with 5% CO_2_. Differentiation promoting effects of IL-4 were saturating at concentrations above 316 U/ml [(18) and data not shown].

### Flow cytometry

For flow cytometry experiments, 200 μL wells containing 10^4^ cells were cultured in triplicates across 96 well flat-bottomed plates. Blimp-1^+/GFP^ and wild type cells stimulated with IL-4 and 10, 2.5, or 0.625 μg/mL of anti-CD40 antibody, or IL-4 alone were harvested periodically for flow cytometry analyses (BD FACSCantoII). Propidium iodide (PI, 0.5 μg/mL final, Sigma cat#287075) for dead cell exclusion and 5,000 beads (Sphero Rainbow Calibration particles [6 peaks] 6.0–6.4 μm, BD Biosciences, cat#556288) for cell counting were added just prior to sample acquisition.

### Cell sorting and long-term live cell imaging

For filming, 5 mL cultures (2 × 10^5^ cells/mL) of cells stimulated with IL-4 and 10, 2.5, or 0.625 μg/mL of anti-CD40 antibody were harvested 85 h after stimulation. Cells were labeled for expression of IgG1 (clone X56, BD Pharmingen cat#550874), and sorted (BD FACSAriaIIu), for generation four cells that were undifferentiated (Blimp-1-GFP^−^) and unswitched (IgG1-APC^−^), to ensure that cells with a similar starting phenotype were tracked and compared in each CD40 stimulation condition.

Sorted cells were re-cultured in phenol red-free B cell medium with the same stimuli concentrations as prior to the sort, at 5 × 10^4^ cells/mL. For each of the three anti-CD40 conditions, 250 μL of cell suspension was placed into a separate well of a pre-prepared chamber slide (Ibidi, cat#80826) where each well was lined with a polymer imprinted with a microgrid array of cell “paddocks” (Microsurfaces, cat#MGA-050-02) (27). Microgrids were prepared aseptically, rinsed with 100% ethanol for sterilization, then left to dry completely to ensure adherence; before wetting again with ethanol such that B cell medium could be introduced to the hydrophobic grids. Chambers were rinsed 10 times with B cell medium, prior to resting overnight in an incubator to aid the dissolution of any air bubbles. Chambers and polydimethylsiloxane (PDMS) microgrids were exposed to 470 nm LEDs (custom made) for at least 30 min prior to the addition of cells, to photobleach the grids and reduce autofluorescence during imaging. The seeding cell density was determined to yield not more than an average of one cell per paddock for filming.

Microscopy images were acquired using a Zeiss Axiovert 200M widefield inverted microscope, equipped with an incubation chamber (37°C, 5% CO_2_, humidifier), plan-apochromat 20x objective (0.8 n.a.), 0.63x c-mount, and a Zeiss AxioCam MRm (1.4 MP) camera. Fluorescence (GFP) and bright field images were acquired for 141 positions, at 15-min intervals, encompassing 7,896 paddocks for the three culture conditions, across 360 time points over the following 89.75 h. The remainder of the sorted cells were placed into triplicate or duplicate cultures in 96-well plates for a concurrent flow-cytometry time course of events, and were run twice-daily for the duration of the filming experiment as a parallel control.

### Single cell fates were manually tracked with visual cues and fluorescence thresholding

For consistent tracking of imaged cells, fluorescence images were first processed using the pipeline reported by Duffy et al. (26). All images from the GFP channel were corrected for uneven illumination of the microscopy stage, thresholded for fluorescence, and binarized to produce an objective indicator of GFP positivity. The image processing method is automatic, and threshold values were computed relative to background illumination using intensity histograms for each image. Resultant images were cropped into individual paddocks, and the processed GFP, unthresholded GFP, and bright field images with GFP overlay for each paddock were concatenated for ease of viewing and stacked into time-lapse films.

Paddocks with individual, undifferentiated cells were identified, and those observed to divide were followed to record the fates of paired offspring. Bright field images were used to manually track cells using their location, size, shape, granularity and trajectory. These properties allowed for the reliable identification of division, as well as death. Shortly before division, cells appeared to lose adhesion and formed large spheres, before cleaving into two smaller cells that do not immediately produce pseudopodia. For death, cells sharply increased in granularity and the circumference of the membrane appeared ruffled, likely due to blebbing, several frames before cell fragmentation was observed, or the membrane perforated and the cell swelled from osmotic intake. This first change in texture was recorded as the cell death time.

For identifying differentiation to ASC, thresholded and binarized images in the GFP channel were followed as a reporter for Blimp-1 expression. Dim light settings are required for extended imaging to avoid phototoxicity, hence chosen voltage and exposure settings also allowed some noise to be detected above the low threshold, from the autofluorescence of the cells and grids. Consequently, differentiation times were only recorded when the cell's fluorescence remained above threshold for three or more consecutive frames (45 min), and then did not disappear for more than one frame at a time; GFP expression would later brighten and cover a larger area. Unthresholded images from the GFP channel were referenced for noise exclusion, and also used for cell identification and tracking based on differentiation status and level of fluorescence.

Sister cells fates were tracked until they either divided again or died. Some cells survived until the end of filming, or were lost due to falling out of focus, migration away from their paddock, or failure to maintain cell ID—these times were also recorded. Homotypic adhesion prevented the tracking of four or more cells using this experimental design.

### Statistical analysis

Data was processed in Matlab 2017b by custom software utilizing in-built functionality. Pearson's correlation coefficient was evaluated using corr, and the reported confidence intervals (CIs) were determined by Fisher's Transformation. We used Yule's Q, a traditional measure of association between pairs of variables, to quantify association between division and death or differentiation and no differentiation. Its asymmetric CIs were determined from a normal approximation to errors in the logarithm of the Odds Ratio. Non-parametric survival function (i.e., Kaplan-Meier) estimates were made using the censoring option of the in-built function ecdf.

### Parametric model fitting procedure

Custom software utilizing the Optimization Toolbox in Matlab 2017b was used for model fitting. The uncensored distributions were assumed to lie in the class of log-normal distributions. For all stimulation conditions, there was a single log-normal time to death T_death_ parameterized by a mean μ_death_ and covariance $\sigma_{\text{death}}^{2}$. For all stimulation conditions, there was a probability, p_diff_, that the differentiation process is active in the cell whereupon it occurs at a log-normally distributed time with parameters μ_diff_ and $\sigma_{\text{diff}}^{2}$. If the process was not on, then the differentiation time was set to be +∞. For each of the three concentrations of 1C10 (0.625, 2.5 and 10 μg/mL), labeled j in (1, 2, 3), it was assumed there was a distinct probability, ${\text{p}}_{\text{div}}^{\text{j}}$, that the division process is active in the cell whereupon it occurs at a concentration-dependent log-normally distributed time with parameters $\mu_{\text{div}}^{\text{j}}$ and $\sigma_{\text{div}}^{2,\text{j}}$. If division was not active, the time was set to be +∞.

For θ = (μ_death_, $\sigma_{\text{death}}^{2}$, p_diff_, μ_diff_, $\sigma_{\text{diff}}^{2}$, ${\text{p}}_{\text{div}}^{1}$, $\mu_{\text{div}}^{1}$, $\sigma_{\text{div}}^{2,1}$, ${\text{p}}_{\text{div}}^{2}$, $\mu_{\text{div}}^{2}$, $\sigma_{\text{div}}^{2,2}$, ${\text{p}}_{\text{div}}^{3}$, $\mu_{\text{div}}^{3}$, $\sigma_{\text{div}}^{2,3}$), a function was written that numerically calculates the likelihood of generating a data point d ∈ D given that parameterization. For example, if a time-lapse frame is taken every h units of time, for a data point d ∈ D in which a cell in stimulation condition j is observed to differentiate in the frame number f_diff_ and undergo death in the frame number f_death_, the likelihood of generating that data point d given the model parameterization θ is

$$\begin{array}{l} \text{L}(\text{d}|\theta )=\text{P}({\text{f}}_{\text{diff}}\;\leq \;{\text{T}}_{\text{diff}}/\text{h}<{\text{f}}_{\text{diff}}+1)\text{P}({\text{f}}_{\text{death}}\;\leq \;{\text{T}}_{\text{death}}/\\ \text{h}<{\text{f}}_{\text{death}}+1)\text{P}({\text{T}}_{\text{div}.}^{\text{j}}/\text{h}>{\text{f}}_{\text{death}.}), \end{array}$$

where the cumulative distribution functions were evaluated using Matlab's logncdf. For a set D composed of the fates of stochastically independent cells, the likelihood of generating the set is the product of the likelihoods of generating each point in the data

$$\begin{array}{l} \text{L}\left(\text{D}|\theta \right):=\Pi_{\text{d}\in \text{D}}\text{L}\left(\text{d}|\theta \right). \end{array}$$

We used Matlab 2017b's Optimization Toolbox function fmincon to identify the maximum likelihood model parameters θ that would generate the data:

$$\begin{array}{l} \theta_{\text{MAP}}:={\text{argsup}}_{\text{θ}}\text{L}\left(\text{D}|\theta \right). \end{array}$$

As sibling cells have correlated times to fate, they are not independent and so the function given above does not describe their likelihoods. Despite that, assuming symmetry in the joint underlying distribution of times to each fate of siblings, the maximum likelihood marginal parameters are obtained by optimizing over the same objective function given above computed on all data, including siblings.

### Reshaped distributions

Competition and censorship alters the underlying distributions of times to differentiation, division and death into those that are observed. For example, the observed marginal probability density function for division under stimulation condition j is related to the uncensored distributions for division and death through the following equation:

$$\begin{array}{l} \text{dP}({\text{T}}_{\text{div}}^{\text{obs},\text{j}}\leq \text{t})/\text{dt}=\text{dP}({\text{T}}_{\text{div}}^{\text{j}}\leq \text{t})/\text{dtP}({\text{T}}_{\text{death}}>\text{t})/\\ \int \text{dP}({\text{T}}_{\text{div}}^{\text{j}}\leq \text{s})\text{P}({\text{T}}_{\text{death}}>\text{s}), \end{array}$$

which differs from the uncensored density of ${\text{T}}_{\text{div}}^{\text{j}}$. Similar expressions hold for ${\text{T}}_{\text{diff}}^{\text{obs},\text{j}}$ and ${\text{T}}_{\text{death}}^{\text{obs},\text{j}}$. Rather than perform numerical integrals to evaluate these, a Monte Carlo approach was taken where 10^6^ samples were drawn from the uncensored distributions parameterized by θ_MAP_, censoring rules were applied to the sampled values, and the resulting empirical distribution functions and densities of the observed variables were determined.

## Results

### Population-level generation-based rate of differentiation is increased by weak CD40 stimulation

**Figure 1:**
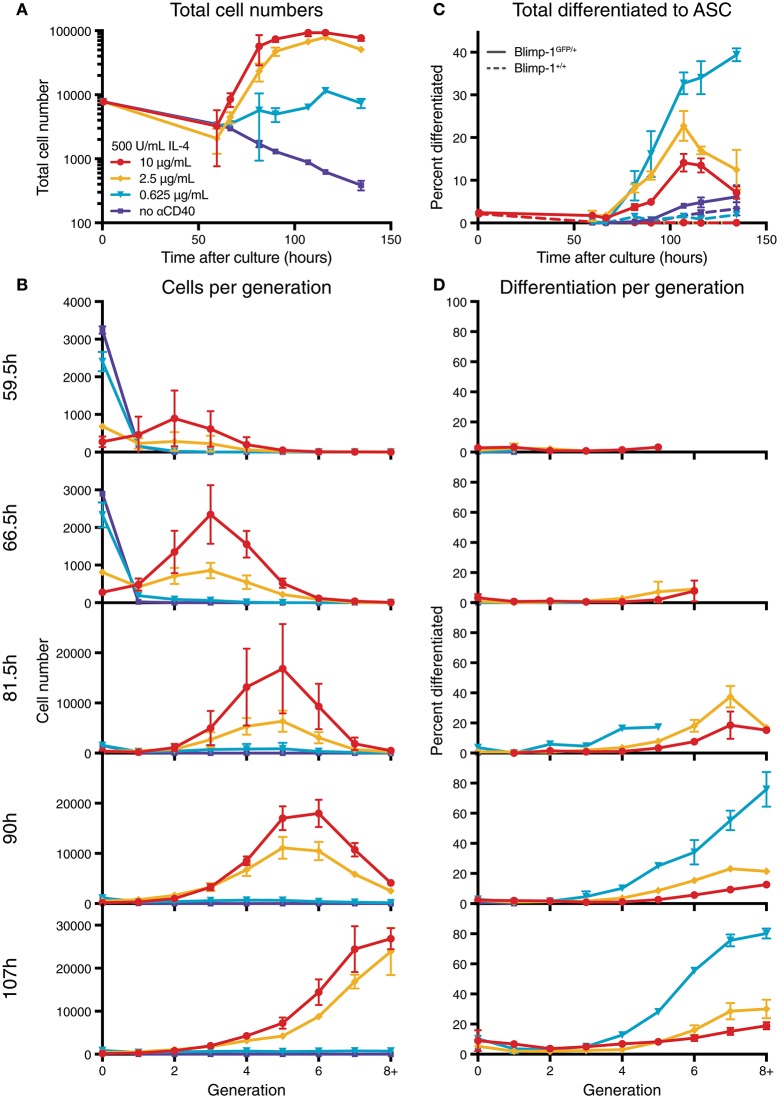
Anti-CD40 concentration alters division and differentiation rates. CTV labeled resting Blimp-1^gfp/+^ B cells were cultured in 500 U/mL IL-4 and 10, 2.5, 0.625, or 0 μg/mL anti-CD40 and harvested over time for flow cytometry analysis. **(A)** Total cell numbers over time. **(B)** Total cells found in each generation. **(C)** The proportion of total viable cells also GFP^+^ (ASC). Dashed lines are from equivalent wild-type control cells used to set GFP gates. **(D)** The proportion of cells in each generation that were GFP^+^. Data points are mean of triplicate cultures ± SEM, and representative of several repeated titration experiments.

To determine the effect of modulating CD40 signal strength, we utilized B cells from Blimp-1-GFP reporter mice to indicate expression of the ASC differentiation program within plasmablasts. In this system, cells expressing GFP secrete Ig at high efficiency (22). Purified resting naïve B cells from reporter mice were labeled with CellTrace Violet (CTV), and equal numbers of cells were placed in culture with varying concentrations of anti-CD40 agonist antibody (clone 1C10) (28) and saturating IL-4 (500 U/mL), and harvested over time (Figures S1, 1). As expected, increasing concentrations of CD40 led to greater cell numbers and increased progression through consecutive generations (Figures S1A,B). Furthermore, division-linked effects on ASC development were apparent as a greater proportion of cells produced Blimp-1-GFP in the advanced generations (Figures 1C,D), consistent with published findings (18). These data also confirmed the observation of Hawkins et al. (29) that lower CD40 stimulation levels resulted in a greater proportion of ASC per generation when compared to equivalent generations in cultures with high CD40 stimulation (Figures 1C,D). An increased rate of differentiation was also measurable in the population as a whole (Figure 1C). Thus, modulating CD40 stimulation strength has two distinct effects on the B cell response: high concentrations promote increased proliferation, whilst low concentrations increase the rate of observed differentiation events per generation. To identify the mechanism that alters cell differentiation by changes in stimulation strength, we undertook direct observation by live imaging.

### Long-term imaging allocates fate assignments for single cells

**Figure 2:**
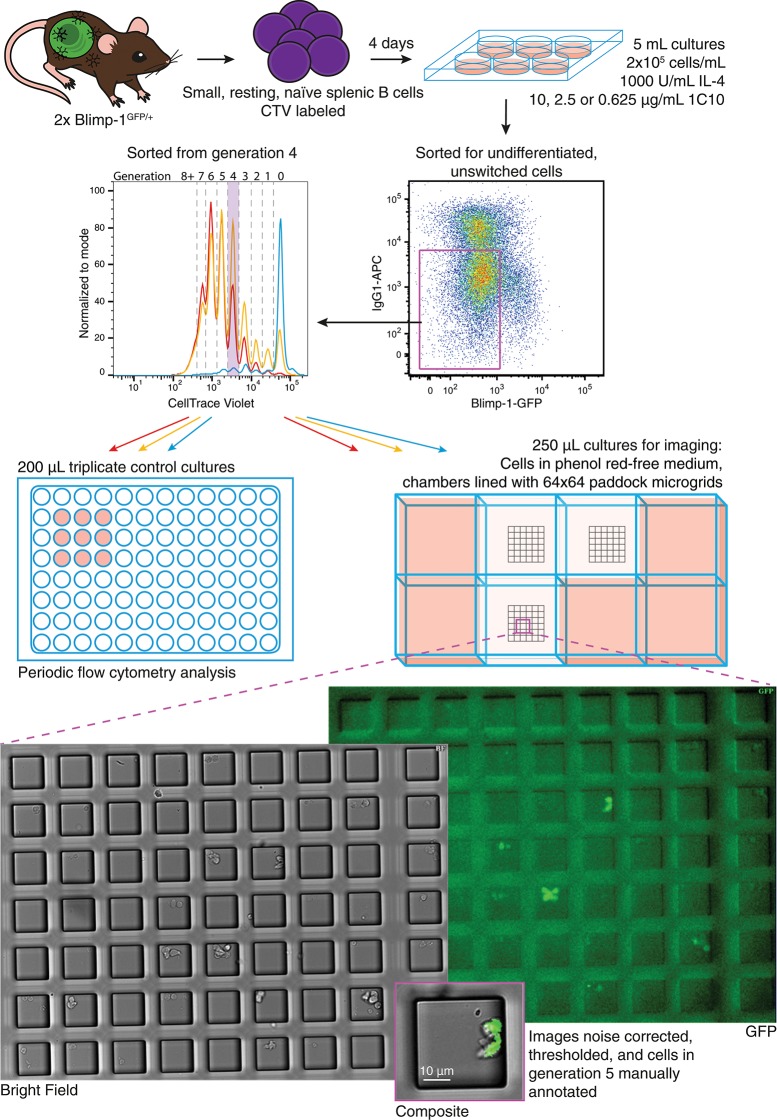
Schematic of live cell imaging experiment. CTV labeled Blimp-1^gfp/+^ B cells were incubated in two stages. Upper panels indicate the initial bulk culture at multiple anti-CD40 concentrations. After 85 h, cells were harvested, labeled with anti-IgG1, and unswitched, GFP^−^ cells identified and sorted from generation 4. Cells were returned to stimulation conditions identical to that prior to sorting, and a portion plated into control cultures for flow cytometry. For microscopy, phenol red-free media was used, and cells from each culture condition were placed into a separate chamber of an imaging slide, each lined with a microgrid of 50 μM cell paddocks. Once settled, the cell density was such that, on average one cell would randomly deposit within one paddock. Media containing pH indicator was placed in surrounding wells to buffer for evaporation and monitor CO^2^ availability. Microgrids were tiled and imaged at 15 min intervals for 89.75 h. Acquired bright field and fluorescence images were processed and manually annotated to record cell fate.

B lymphoblasts stimulated by CpG can be tracked individually through multiple cell generations (30, 31). In contrast, observing differentiation by live imaging following CD40 stimulation is challenging due to homotypic adhesion. The development of cell aggregates restricts tracking of individual progenitor cells for more than one or two generations (30). A new method was introduced in Duffy et al. (32) to circumvent this problem. By harvesting and disaggregating CTV labeled, proliferating B cells after a few days in culture, then seeding sorted, individual cells into microgrids to maintain segregation, (27) cells from different generations were observed to divide and their progeny followed until their next fate (32). Here we adapted this protocol, illustrated in Figure 2, to observe the effect of CD40 stimulation strength changes on differentiation and division times, as well as concordance in sibling fates.

In initial bulk cultures CTV labeled Blimp-1-GFP reporter B cells were incubated with IL-4 and varying concentrations of anti-CD40 (10, 2.5, 0.625, and 0 μg/ml) for 4 days, resulting in the expected variation in division and differentiation rates (Figure S1A). To compare the subsequent fate of undifferentiated cells from the same generation, cells from each culture were sorted by flow cytometry for those in generation 4, and seeded into 250 μL chambers containing microgrids for a further 90 h of live cell imaging (see Materials and Methods). Control cell cultures were prepared in parallel at the same density in 96 well plates, and triplicate 200 μL cultures were analyzed periodically to ensure the overall population response of the sorted cells was consistent (Figure S2). Control analyses indicated that sorted cells were GFP^−^ and in generation 4 at the time they were re-cultured (Figure S2B). These cultures also confirmed that cells stimulated with higher concentrations of anti-CD40 divided faster, resulting in greater CTV dye dilution (Figure S2B), and higher total cell numbers (Figure S2C). Despite the variation in progression through generations, a greater proportion of cells in 2.5 μg/mL anti-CD40 were GFP^+^ than in 10 μg/mL (Figure S2D). As these proliferation and differentiation features were consistent with earlier studies, manual tracking and analysis of the parallel single cell imaged cultures were undertaken.

### Single cell data recapitulated fate changes seen at population level

**Figure 3:**
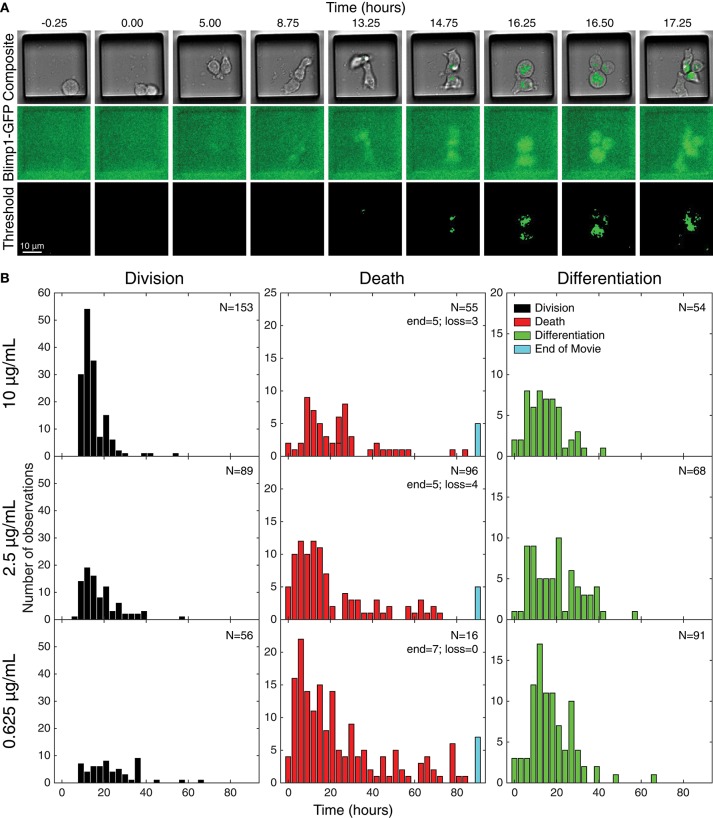
Tracking individual cell fates over time. **(A)** Example microscopy images from one cell paddock, with selected frames from the bright field and GFP channels over time. Sorted cells (generation 4) observed to divide (generation 5) and, if still GFP^−^, were tracked further to record subsequent division, death, and differentiation times. The initial mitotic event was assigned time = 0 in subsequent analyses. Division and death were visually discernible in the bright field channel (top row). Fluorescence images in the GFP channel (middle row) were corrected for uneven illumination, thresholded, and binarized for annotating differentiation to ASC (bottom row). **(B)** Histograms of the total data for each fate recorded, timed from first observed cell division and allocated to 3 h bins. Number of recorded cells indicated in panels (*N*). Cells that reached the end of the imaging period and had neither divided nor died were recorded as “end” and appear in the blue bar. Number of lost cells also indicated.

Acquired images were processed and thresholded to facilitate GFP scoring, as described in Methods and illustrated in Figure 3A. After initial visual inspection, “paddocks” identified with single cells that undertook their first division as GFP^−^ cells were selected, and the resulting two siblings followed manually, to record their times to changes in fates (Figure 3). For the data presented here, the time of the cell's first division (therefore from generation 4 to 5) is set as time 0, the initiating event time, and the siblings being tracked are in generation 5. The complete annotated data set was converted to such times by calculating the times between the first observed division and subsequent fates (differentiation to ASC, division or death). Histograms of these times are plotted in Figure 3B, illustrating the heterogeneity in each outcome.

**Figure 4:**
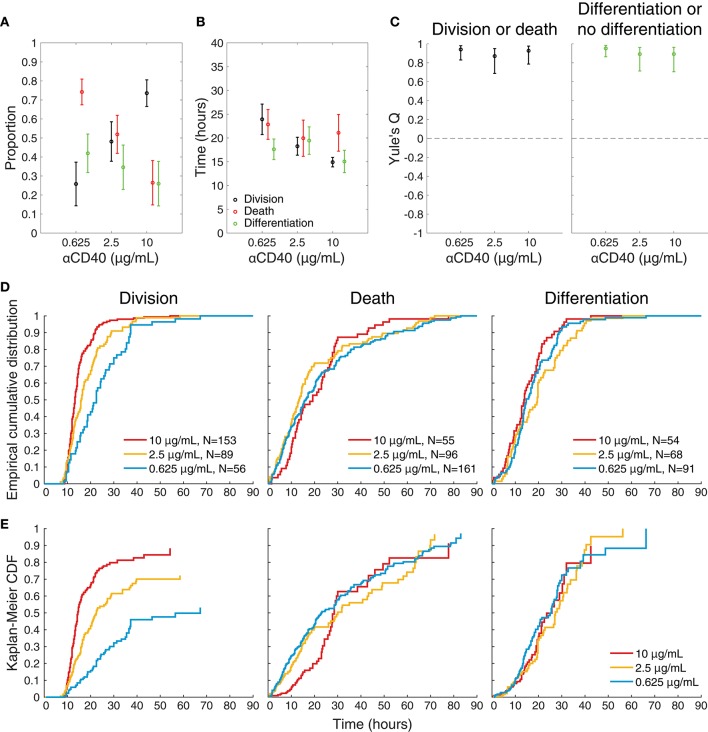
Features of single cell imaged data. **(A)** The proportion of tracked single cells that underwent each fate. Fisher's exact test was performed to compare proportions of cells to undergo fates between 0.625 vs. 2.5, 0.625 vs. 10, 2.5 vs. 10 μg/mL anti-CD40. Division vs. death: *p* = 4.18 × 10^−6^, 1.77 × 10^−23^, and 3.01 × 10^−7^, respectively. Differentiation vs. no-differentiation: *p* = 0.15, 0.0007, and 0.078, respectively. **(B)** For cells reaching each fate the average time is shown with 95% CIs. Kruskal-Wallis test was performed to compare the times to fates between different anti-CD40 concentrations. Division: *p* = 3.8741 × 10^−8^, death: *p* = 0.2386 and differentiation: *p* = 0.1354. **(C)** Yule's Q, a measure of concordance in fate, shows that sibling fate selection (death or division, differentiation or no differentiation) is highly symmetric at all anti-CD40 stimulation levels with 95% CIs indicated by bars. **(D)** Cumulative frequency distributions of raw data for time to each fate. **(E)** Uncensored times to fate as determined by Kaplan-Meier survival function estimates overlaid for each anti-CD40 concentration. Division was uncensored from the influence of death, death was uncensored from division, and differentiation was uncensored from both division and death. Data from all tracked cells are included.

To visualize differences between culture conditions, observed proportions to undergo each fate are shown in Figure 4A, and mean times to reach each fate in Figure 4B. These data suggest that cells stimulated with high concentrations of anti-CD40 were more likely to divide and less likely to die (Figure 4A), and when they did divide they completed mitosis more quickly (Figure 4B) The proportion of cells observed to differentiate is also consistent with flow cytometry time courses, in that a higher proportion of cells differentiated to ASC with lower CD40 stimulation (Figure 4A). Thus, despite segregation into cell paddocks by the microgrids, the filmed cells recapitulated the fate outcomes measured by flow cytometry at the population level.

### Stimulation strength does not affect sibling correlations or concordance

Whether stimulation strength affected differentiation by influencing asymmetry in fate was first assessed. For each of the three concentrations (0.625, 2.5, and 10 μg/mL, respectively) 78, 68 and 75% (±8, 9, 8% as 95% CIs) of siblings take the same differentiation or no differentiation and death or division fates. Figure 4C plots Yule's Q, a measure of concordance for opposing fates (division vs. death, and differentiation vs. no differentiation) relative to their frequency of occurrence in the population. The consistent, high values of Q indicate the significant concordance found for both division-death and differentiation-no differentiation fates of siblings was not affected by CD40 stimulation strength. Thus, strong sibling concordances and correlations were found in this experiment, in line with earlier findings. Interestingly, these sibling similarities did not appear to be controlled by altered CD40 stimulation strengths, despite the marked changes in division times, and differentiation rates.

### Uncensoring cell fate time distributions

Having eliminated modulation of asymmetric fates as a control feature regulated by anti-CD40 concentration, we turned to the theory of competing fates as a potential driver of heterogeneity. Under this hypothesis, autonomous processes leading to each fate are underway within the cell. The order in which they complete determines the fate that the cell is observed to take. As observed times to fate are heterogeneous, the mathematical framework of probability is necessary to describe them. It encapsulates the heterogeneity irrespective of whether its source is truly stochastic processes within each single cell, or arises as a result of unidentified heterogeneous lineage properties. The hypothesis suggests that the apparently complex correlation structures observed in cell fate data are a consequence of observed times to fate being the product of competition and censorship, and leads one to query the role of external regulation on each of the autonomous processes (26, 32).

Figures 4D,E shows the result of applying the standard non-parametric survival function estimator, the Kaplan-Meier estimator (33), to the raw cumulative frequency data for each fate (Figure 4D) to reveal the pre-competition, uncensored time-to-fate distributions (Figure 4E), assuming probabilistic independence of these underlying timed mechanisms. For these plots division is assumed to censor death, death censor division, and both division and death censor differentiation. In some instances, the remaining proportion is >0 (i.e., plot does not reach a height of 1), indicating the observation time was too short to capture all possible events in this category; or alternatively that the remaining proportion of cells were incapable of undergoing that fate.

Within this competing timers model, these results are consistent with the hypothesis that CD40 stimulation strength had a significant impact on cell division times with little direct effect on the underlying distributions of times to death or differentiation. Higher levels of anti-CD40 reduces division times, positively impacting the proportion of cells in the population that progress to the next generation, while reducing the proportion of cells that differentiate. Together, these results are consistent with the hypothesis that CD40 stimulation controls the time to divide, but not the times to differentiate or die.

### Parametric model based on competition and changes in division time only

Under the stochastic competition hypothesis, not all processes need to be in operation in every cell. In particular, there is a possibility that neither differentiation nor division partake in the competition. To extract these propensities to differentiate and divide (i.e., likelihood that the underlying division or differentiation machinery is active within a cell), we created a parametric statistical model by assuming that underlying time-to-fate distributions are log-normal given fate is in operation, but with a possibility that differentiation and division are inactive (discussed in Materials and Methods). Based on the observations from application of the Kaplan-Meier estimator, we assumed that the uncensored differentiation and death distributions were unchanging with stimulus and that only the division distribution and the propensity to divide were altered. This resulted in a single three-parameter description for differentiation (mean, variance, propensity to differentiate) and a two-parameter description for death (mean, variance) across all three stimulation conditions, along with a three-parameter description for division (mean, variance, propensity) per stimulation condition.

**Figure 5:**
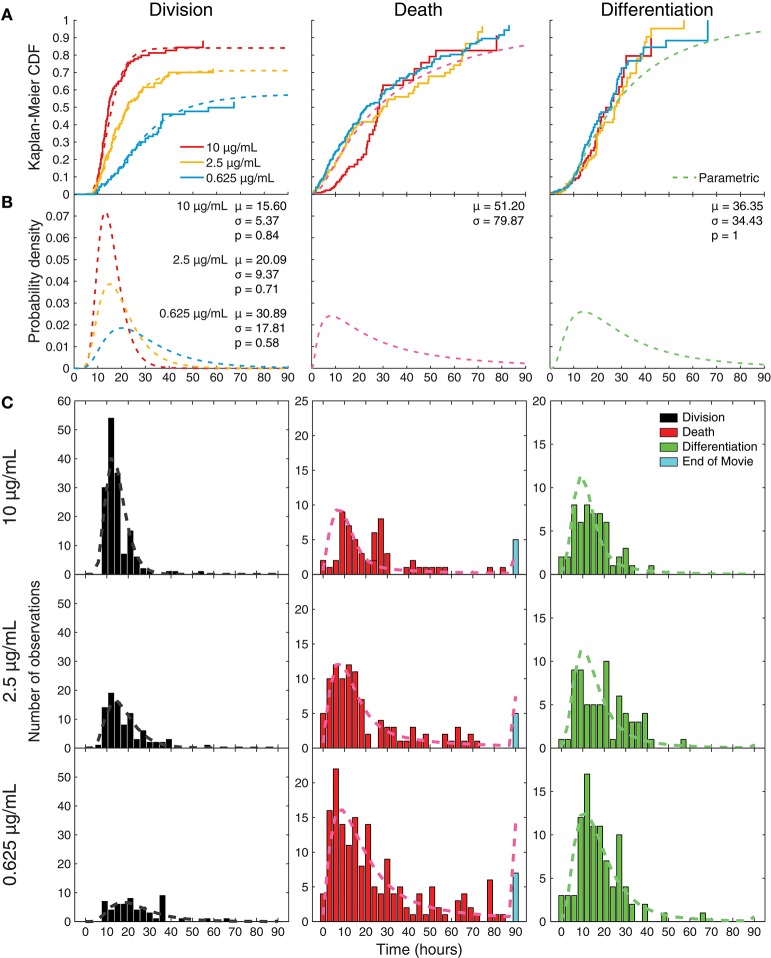
Parametric model fitting with constrained parameters to censored data. **(A)** Maximum likelihood parametric model-fits to the data, under the constraints that death and differentiation time distributions were the same for all conditions, while the division time distribution and propensity for division could change with CD40 stimulation; overlaid with Kaplan-Meier analyses. **(B)** Uncensored parametric plots drawn as probability density functions; mean division times (μ) get longer with less CD40 stimulation, as variance (σ) increases and propensity (p) to divide decreases. **(C)** Predicted outcomes calculated from these constrained parametric distributions overlaid onto the observed data.

The uncensored lognormal curves with the highest likelihood of fitting to the data are shown in Figure 5A for comparison with the per-condition non-parametric uncensoring. The parametric and non-parametric estimates are in good agreement, further supporting the hypotheses of the parametric model. Having ascribed lognormal curves to the probability distributions of times to fate, fitted parameters from different conditions could be compared for deviations in means, variations, and probabilities (Figure 5B). For decreasing anti-CD40 concentrations (10, 2.5, 0.625 μg/mL), the model fit division time distributions to the data with increasing mean (15.60, 20.09, 30.89 h) and variance (5.37, 9.37, 17.81 h), while the propensity for division being “on” in the cell decreased (0.84, 0.71, and 0.58, respectively). Death times (mean 51.20 h, variance 79.87 h) and differentiation times (mean 36.35 h, variance 34.43 h, propensity 1) were fitted with one distribution each for all of the CD40 stimulation concentrations, with the assumption that the probability of death is always ultimately 1. The best-fit propensity for differentiation was 1, suggesting that differentiation is always “on” in a stimulated cell and so is the default action that occurs when either division or death does not censor it. Overlaying the raw data, Figure 5C provides the extrapolation from these best-fit uncensored probability distribution functions to what they predict would be observed as a consequence of competition and censorship. The model predicts that a small proportion of cells will take their fate, typically death, after cessation of filming. A small number of cells are, indeed, observed to have neither died nor divided by the end of the microscopy session, and these are plotted at the end of the death-time histograms, according well with the out-of-sample model prediction.

### Further features of the data consistent with competition

**Figure 6:**
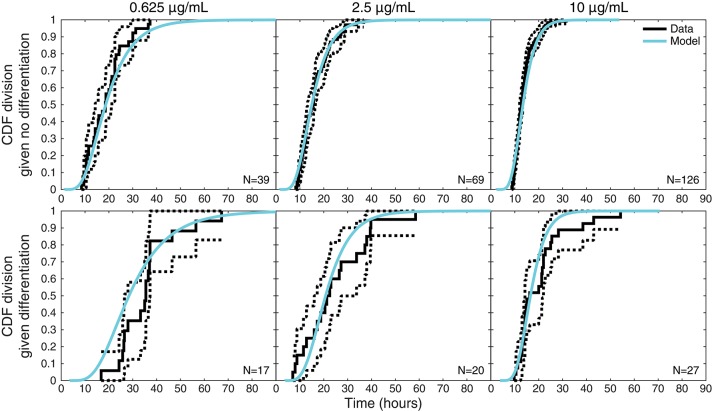
Times to divide given differentiation or not. For each stimulation condition, upper panels display the empirical distribution of times to divide for cells that did not differentiate (solid black lines) with 95% confidence intervals (dotted black lines) computed with Greenwood's Formula. The lower panels display the same information for cells that were observed to differentiate before dividing. Also shown on both are the model-predicted distributions (cyan) of times to divide conditional on a cell differentiating or not, which differ as a consequence of the reshaping by competition.

Figure 6 plots an additional interesting feature of the data that might appear to require an involved explanation. The upper panels display the empirical cumulative distribution of the times to divide of cells that did not differentiate for each stimulation condition, while the corresponding lower panels display the times to divide for cells that were observed to differentiate. For each condition, these two distributions are distinct, with division times being—on average—longer for cells that differentiated. The equivalent plots for times to death can be found in Figure S3, where the same phenomenon is exhibited.

Outside of the competition model, this seems to suggest distinct distributions for times to division or death fates dependent on whether a cell differentiates or not. Within the competition hypothesis, however, it is instead an intrinsic consequence of the model structure. Also displayed in the upper panel of Figure 6 is the conditional distribution of the time to divide given that the cell did not differentiate as determined by the model, while the lower panel displays the model's conditional distribution of the time to divide given a cell did differentiate. Within the competition model, these two observed distributions are expected to be distinct as a consequence of censoring: knowing that a cell differentiates ensures a lower bound on the time to divide, conditioning it to be larger. Thus, the competition model inherently anticipates and accounts for these apparently involved features of the data within one mechanistically simple hypothesis that would otherwise require a model with significantly more parameters to explain.

## Discussion

The B lymphocyte is an ideal model system for studying cell fate control, being highly tractable to *in vitro* manipulation and sensitive to many alternative receptor driven signals that alter its behavior. Here we adopted this system and sought an answer to how varying the strength of one significant signal, transmitted through CD40 on the B cell surface, could affect the rate of development to ASC. This question is of particular interest as earlier studies noted the effect to be “paradoxical”: weaker stimulation leads to a greater rate of differentiation with each generation (29). Building on previous filming experiments (26, 30), chamber slides were used to sustain and image cells in varying levels of anti-CD40 stimulation. Miniature paddocks segregated the individual cells and reduced interference from homotypic adhesion (27). These data consisted of the recorded times to fates of hundreds of sibling pairs, making it suitable for quantitatively challenging hypotheses.

Analyzing these data allowed the elimination of the hypothesis that the paradoxical differentiation effect was due to changes in asymmetric cell division, as siblings had a high concordance in fate that was unaffected by stimulation strength. Our attention turned to ask whether the theory of competing fates (26, 32) could explain the phenomena. We hypothesized that increased differentiation could arise by cells transitioning faster within each generation, or by division times slowing, and thus allowing cells more time to differentiate before they divided again. Consistent with the latter hypothesis, the data, when uncensored, revealed that CD40 stimulation level regulated division times and division proportions, but had no effect on either differentiation or death times. Parametric fitting to differentiation times only required one set of parameters (mean, variance, propensity), whereas constraining division times to a single set of parameters would have produced poorly fitted outcomes. Hence, assuming independently timed control of fates, we were able to reject the hypothesis that CD40 stimulation was controlling either the proportions of cells capable of differentiating to ASC, or the time required for cells to differentiate. Instead, changes in division times were sufficient to re-create the differentiation patterns seen with flow cytometry.

This analysis supported the hypothesis that low CD40 stimulation slows division times, and consequently allows more time for cells to differentiate. Thus, regulation of division time by stimulation strength is identified as a controlling feature of fate decisions by the stimulated B cell. An influence of cell cycle length on differentiation has been noted in other cell systems suggesting this may be a biologically widespread regulatory mechanism (34, 35). We also assume the likelihood of differentiation is dependent on, and in turn altered by, the concentration of cytokines in culture. Further experiments will be required to assess this possibility.

These studies raise the question of how signals and internal molecular processes alter the time to different fates. One mechanism reported to date posits accumulation of transcriptional regulators. Kueh et al. (36) imaged hematopoietic progenitor cells and noted the choice between becoming a macrophage-lineage cell or a B cell was dependent upon the timed accumulation of transcriptional regulator PU.1. The longer cells took to divide, the more likely they were to accumulate the higher levels of PU.1 needed for macrophage commitment. A variant of this mechanism requiring both timed production and loss was noted by Heinzel et al. (37) for expression of Myc as a controller of division progression. Myc accumulated in proportion to signal strength and decayed at a constant rate, independently of division, eventually leading to a timed cessation of mitosis. As transcriptional regulators are important drivers for many fate changes, equivalent timed changes in expression level based on accumulation are likely to be in operation in mature B cells. Further experiments measuring levels of transcriptional regulators and correlating levels with fate outcomes in individual cells will be informative to identify this and other putative molecular mechanisms (15).

**Figure 7:**
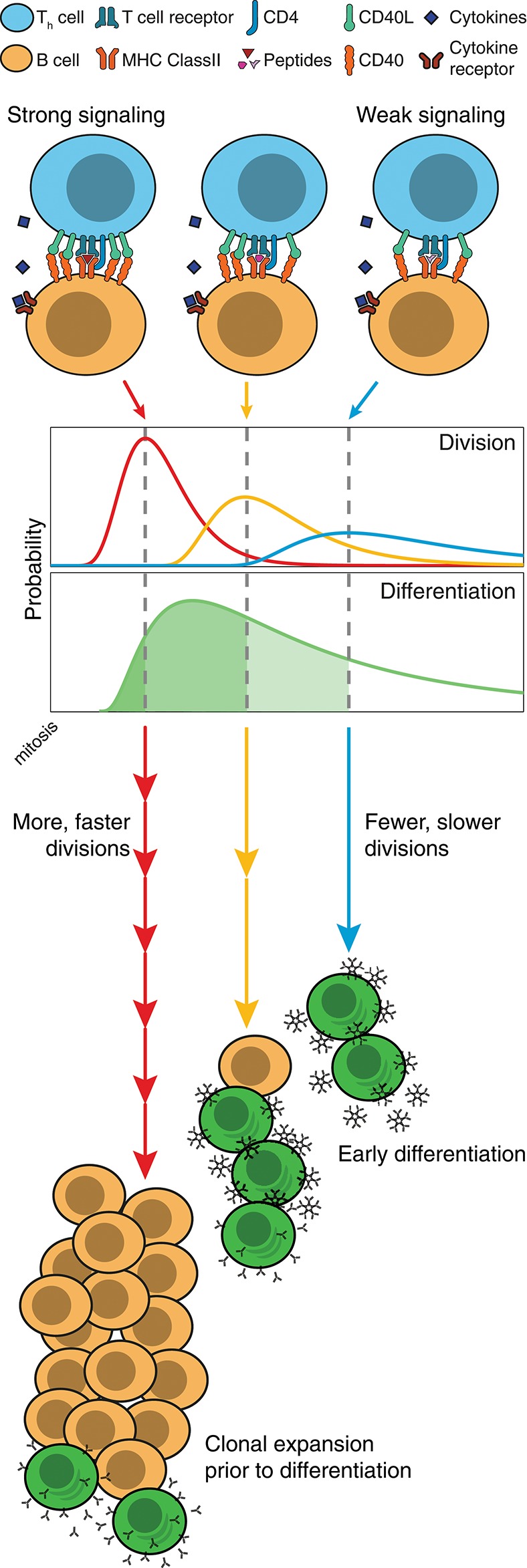
Model of division-based control of B cell fate. Quantitative differences in CD40 signaling leads to proportional differences in division times, as well as the number of divisions completed. Even without modifications to underlying differentiation times, cell populations can differentiate as a result of competing cell fates. With slow divisions, the multitude of weakly stimulated cells automatically differentiate, and rapidly form large numbers of unswitched, low affinity antibody secreting cells. Meanwhile, those cells that receive strong stimulation divide rapidly, and hold back their differentiation mechanism until the clone has undergone expansion and isotype modifications, thus leading to an increased net number of higher affinity plasmablasts.

These findings also offer insights into the controlling systems for antibody generation during a T-dependent immune response. T and B cell cooperation is an active process that occurs in two distinct sites during an immune response. Initial engagement occurs in the extrafollicular zones of lymphoid tissue and leads to the heterogeneous production of antibody secreting plasmablasts, that are typically short-lived and of weak to moderate affinity (38). Effective T and B cell collaboration requires an unbroken sequence of graded quantitative events that begins with antigen capture by the B cell receptor (BCR) and upregulation of T cell costimulatory surface molecules such as CD28, class II MHC and CD40 (39, 40). For effective stimulation by T cells, antigen must also be internalized by the B cell and presented on the cell surface, providing further opportunities for quantitative titration of the outcome (41). These variables in turn determine the level of stimulation received by an engaged T cell and subsequently the level of CD40L expression, and the rate of cytokine production provided to the B cell during the collaborative event (42). While the combinatorial possibilities are large, given the results here, we can identify a key principle in operation: even if holding all other variables constant, quantitative differences in CD40L, as the result of the chain of early events, will lead to proportional variations in average division times as well as the number of divisions completed (43). By slowing division, the weaker cells automatically assume a greater likelihood of differentiating and of dying. The more strongly stimulated cells will divide rapidly, automatically holding back their differentiation to ASC and leading to greater selection and expansion. Based on these findings we suggest that, as stimulated cells lose access to T cells, B cells slow division and automatically transition to secreting plasmablasts, provided cytokines are also present. Thus, the more avid and competent B cells are naturally selected for expansion while their differentiation is suppressed, leading, ultimately, to an increased net number of higher affinity plasmablasts overall (illustrated in Figure 7). This model is consistent with the studies of Paus et al. showing greater proliferation and overall generation of plasmablasts by higher affinity B cells (44). As antibody isotype switching is also strongly linked to progressive division and unaffected by the strength of CD40 stimulation (18, 19), this selection mechanism will, without additional cellular machinery, result in higher affinity clones that transition, automatically, to produce specialized antibody subtypes. It seems likely that the quantitative relation between all of these processes has evolved to provide an optimal balance for generating protective antibody over time.

An important second site of T-B cell collaboration is the germinal center. A subset of activated T and B cells migrate to primary follicles to initiate and sustain this reaction (45–47). At this site B cells undertake successive rounds of somatic hypermutation and selection that generates fully differentiated plasma cells and long-lived high affinity, memory B cells. Selection of B cell clones in the germinal center is critically dependent upon T cells and CD40-CD40L interactions (17, 48, 49). B lineage cells in the germinal center are distinct from those generated *in vitro*, and seen in extrafollicular sites (50). However, labeling studies *in vivo* have determined that the division rate is affected by the strength of stimulation provided by T cell help (51, 52). These observations taken together with the *in vitro* findings of the present paper, imply a general mechanism where the rate of proliferation is linked to, and tempers, the fate of competent, highly stimulated cells to achieve an optimal dynamic outcome with minimal direct control over differentiation.

## Author contributions

JZ, PH, and KD designed all experiments and analyzed and interpreted experiments and wrote manuscript. JZ performed experiments and undertook all data annotation. JM developed the data pipeline, conducted the image processing, and oversaw microscopy setup.

### Conflict of interest statement

The authors declare that the research was conducted in the absence of any commercial or financial relationships that could be construed as a potential conflict of interest.
