# Supplementary material for: Stochastically Timed Competition Between Division and Differentiation Fates Regulates the Transition From B Lymphoblast to Plasma Cell
Source: Front Immunol. 2018 Sep 10;9:2053. doi: 10.3389/fimmu.2018.02053 (PMC6139340; doi:10.3389/fimmu.2018.02053)
Supplement: Supplementary file 1 [file Image_1.pdf]

Supplementary figures

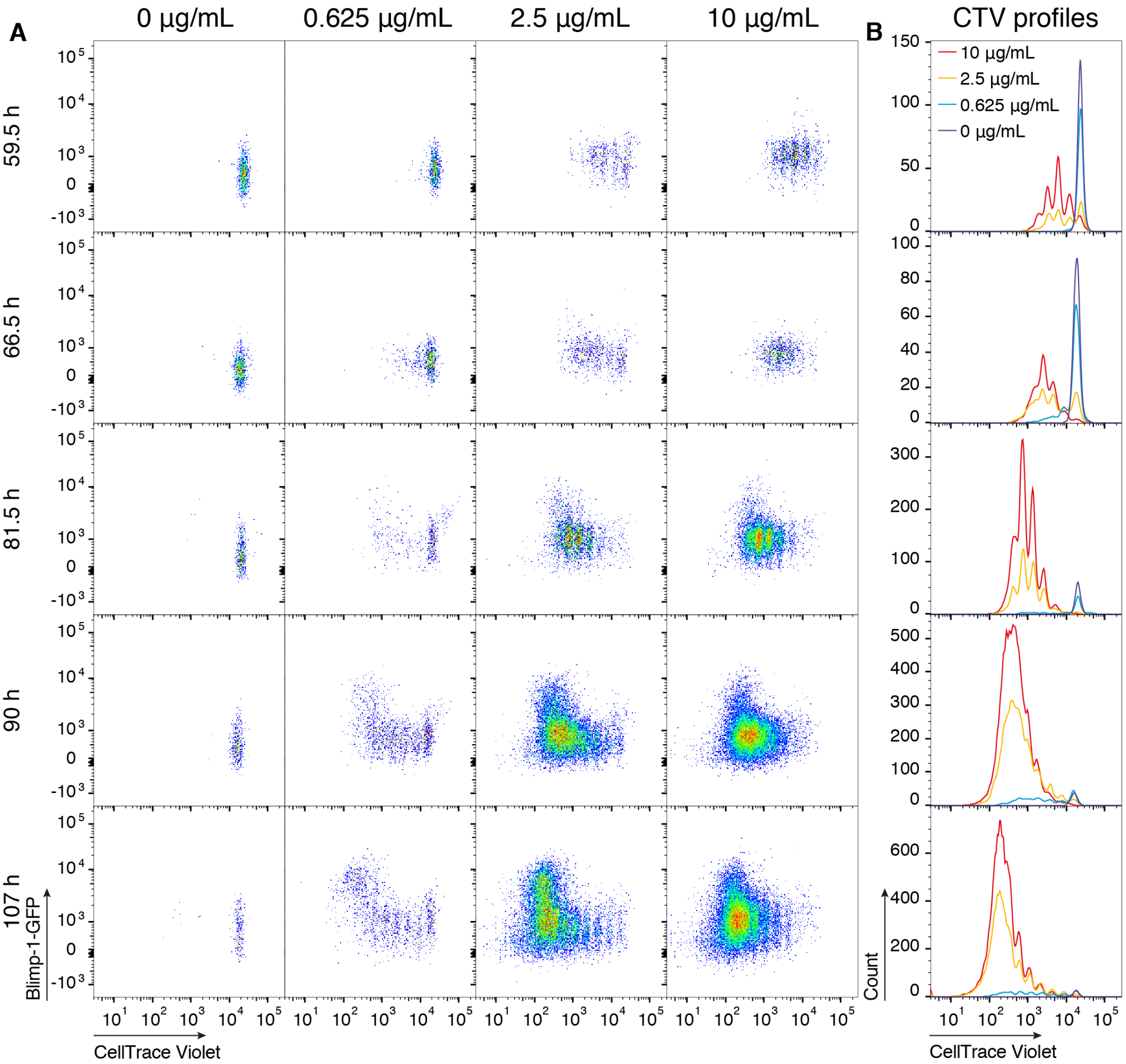

FIGURE S1 | B cell proliferation and differentiation is affected by anti-CD40 concentration. CTV labelled resting Blimp-1$^{gfp/+}$ B cells were cultured in the indicated concentrations of anti-CD40 with constant IL-4 and harvested over time. **(A)** Plots of CTV vs Blimp-1-GFP showing increases in GFP^{+} cell frequency with advancing divisions. **(B)** CTV histograms of total live cells overlaid to compare division progression in response to different anti-CD40 levels. Data representative of multiple CD40 titration experiments.

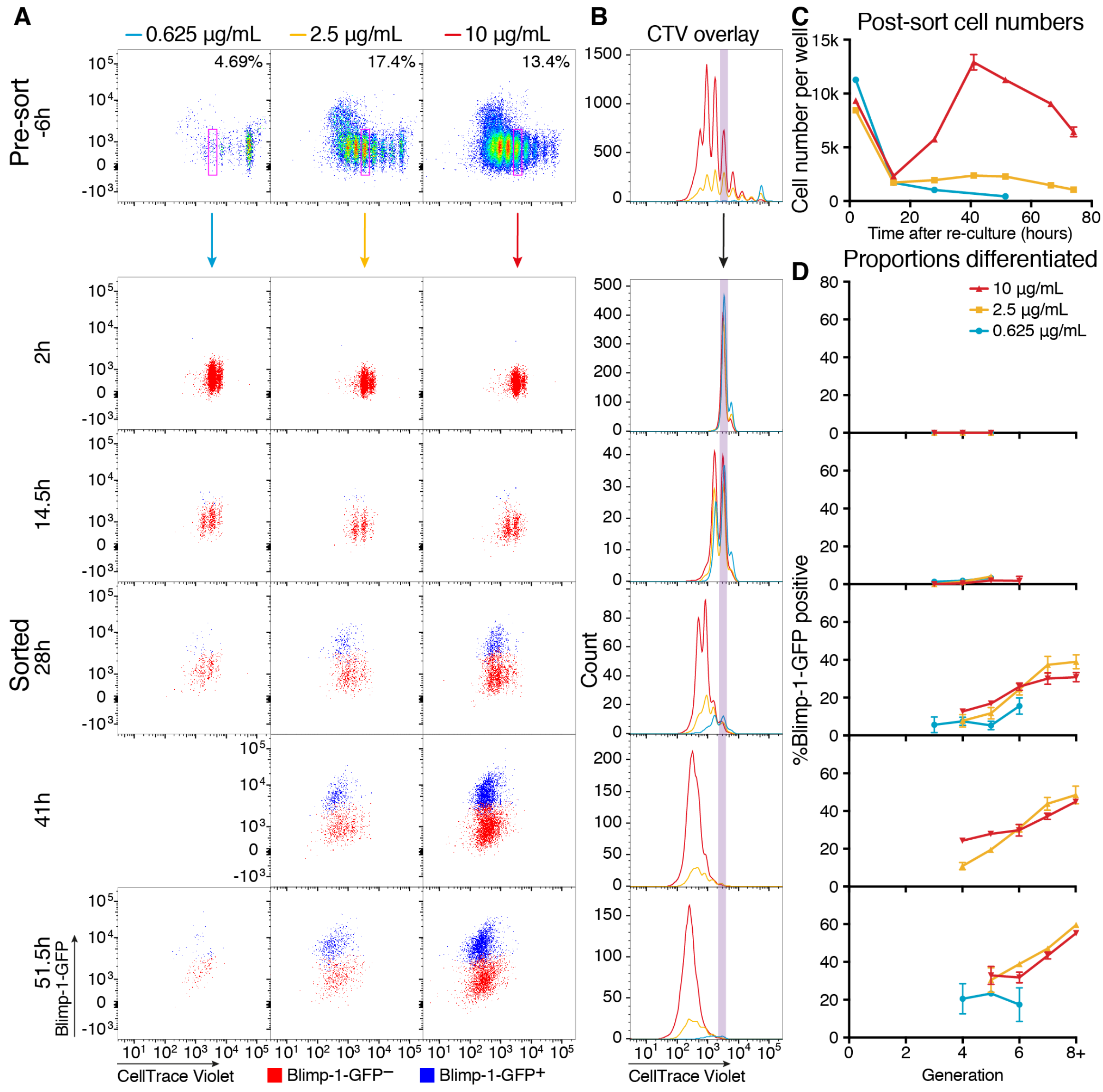

FIGURE S2 | Control cultures for long-term imaging campaign. CTV labelled resting Blimp-1$^{gfp/+}$ B cells were cultured in the indicated concentrations of anti-CD40 with IL-4 and harvested after 4 days. **(A)** Cells analyzed by flow cytometry showing CTV versus Blimp-1-GFP. Pre-sort row (- 6 h) indicates varying proliferation and differentiation in each culture. Sorted cell rows (2 – 51.5 h) show successive harvests of control cells after cytometric sorting of undifferentiated cells from generation 4 (gating box shown in pre-sort dot plot). Blimp-1-GFP^{+} cells indicated in blue. **(B)** CTV histograms overlaid indicating differences in division progression of presorted cultured cells and subsequent control cultures of sorted generation 4 cells. Shaded bar provides reference for generation 4. **(C)** Shows cell numbers recovered over time from re-culture of sorted cells, where ‘0’ is 91 h after initial stimulation. **(D)** The proportion of GFP^{+} cells upon re-culture of sorted cells shows progressive changes with division.

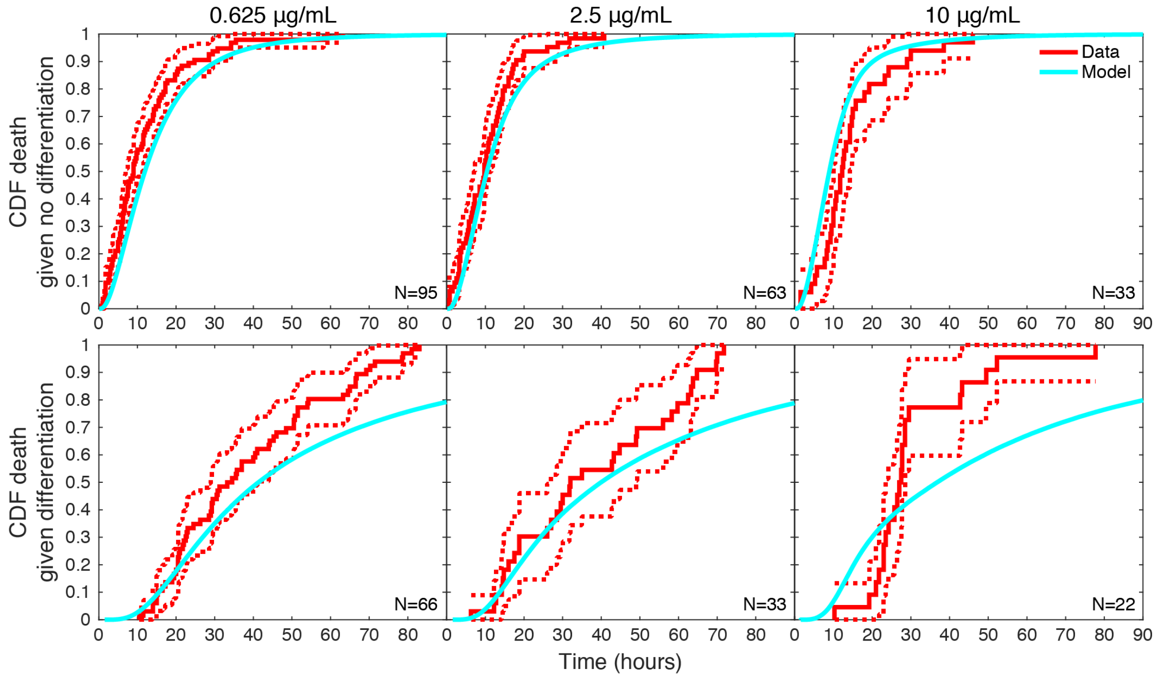

FIGURE S3 | Times to death given differentiation or not. For each stimulation condition, upper panels display the empirical distribution of times to death for cells that did not differentiate (solid red lines) with 95% confidence intervals (dotted red lines) computed with Greenwood's Formula. The lower panels display the same information for cells that were observed to differentiate before dying. Also shown on both are the model-predicted distributions (cyan) of times to death conditional on a cell differentiating or not.
